# GLP-1 Analogues in the Neurobiology of Addiction: Translational Insights and Therapeutic Perspectives

**DOI:** 10.3390/ijms26115338

**Published:** 2025-06-01

**Authors:** Juan David Marquez-Meneses, Santiago Arturo Olaya-Bonilla, Samuel Barrera-Carreño, Lucía Catalina Tibaduiza-Arévalo, Sara Forero-Cárdenas, Liliana Carrillo-Vaca, Luis Carlos Rojas-Rodríguez, Carlos Alberto Calderon-Ospina, Jesús Rodríguez-Quintana

**Affiliations:** 1 Pharmacology Unit, Department of Biomedical Sciences, School of Medicine and Health Sciences, Universidad del Rosario, Bogota 111221, Colombialuisca.rojas@urosario.edu.co (L.C.R.-R.); carlos.calderon@urosario.edu.co (C.A.C.-O.); 2 Research Group in Applied Biomedical Sciences (UR Biomed), School of Medicine and Health Sciences, Universidad del Rosario, Bogota 111221, Colombia; 3 ZONAMEDICA MR S.A.S., Av Kra. 45 No 105-21, Bogota 110111, Colombia; 4 Fundacion CardioInfantil-Instituto de Cardiología, Bogota 111156, Colombia; 5 Hospital Universitario Mayor Mederi, Bogota 111411, Colombia

**Keywords:** glucagon-like peptide-1 receptor agonists, substance-related disorders, brain–gut axis, alcoholism, tobacco use disorder, cocaine-related disorders, amphetamine-related disorders, opioid-related disorders, blood–brain barrier, translational research, biomedical

## Abstract

Glucagon-like peptide-1 receptor agonists, originally developed for the treatment of metabolic disorders, have recently emerged as promising candidates for the management of substance use disorders. This review synthesizes preclinical, clinical, and translational evidence on the effects of glucagon-like peptide-1 receptor agonists across addiction models involving alcohol, nicotine, psychostimulants, and opioids. In animal studies, glucagon-like peptide-1 receptor agonists consistently reduce drug intake, attenuate dopamine release in reward circuits, and decrease relapse-like behavior. Clinical and observational studies provide preliminary support for these findings, particularly among individuals with comorbid obesity or insulin resistance. However, several translational barriers remain, including limited blood–brain barrier penetration, species differences in pharmacokinetics, and variability in treatment response due to genetic and metabolic factors. Ethical considerations and methodological heterogeneity further complicate clinical translation. Future directions include the development of central nervous system penetrant analogues, personalized medicine approaches incorporating pharmacogenomics, and rigorously designed trials in diverse populations. Glucagon-like peptide-1 receptor agonists may offer a novel therapeutic strategy that addresses both metabolic and neuropsychiatric dimensions of addiction, warranting further investigation to define their role in the evolving landscape of substance use disorder treatment.

## 1. Introduction

Addiction, as defined by the fifth edition of the *Diagnostic and Statistical Manual of Mental Disorders* (DSM-5), is a chronic, relapsing disorder characterized by compulsive substance use despite harmful consequences [1]. Substance use disorder (SUD) encompasses cognitive symptoms, including intense craving that impairs concentration and decision-making, behavioral symptoms such as persistent use despite adverse outcomes and the neglect of obligations, and physiological symptoms like tolerance and withdrawal [1]. These manifestations are organized into four diagnostic dimensions: impaired control, social impairment, risky use, and pharmacological criteria. Notably, tolerance and withdrawal resulting solely from medically supervised treatment do not suffice for diagnosis unless accompanied by compulsive use [2]. These criteria guide both diagnostic classification and long-term treatment strategies, given the neuroadaptive changes that increase vulnerability to relapse.

Addiction constitutes a critical public health issue. According to the World Health Organization (WHO), alcohol and drug use accounted for 3.2 million deaths globally in 2019, with 2.6 million due to alcohol and 0.6 million to psychoactive substances [3]. The burden is disproportionately higher among men. Alcohol-related deaths primarily involve non-communicable diseases such as cardiovascular disease and cancer, while opioids, amphetamines, and cocaine were the most lethal among psychoactive substances. Approximately 400 million people worldwide were estimated to have alcohol use disorders (AUDs), with over half meeting criteria for physiological dependence [3].

Conventional SUD management relies on integrated behavioral and pharmacological strategies. Psychotherapies such as cognitive–behavioral therapy, motivational interviewing, and contingency management are well established [4]. Pharmacological options target withdrawal, craving, and relapse prevention and include methadone, buprenorphine, naltrexone, disulfiram, acamprosate, varenicline, and bupropion [5]. However, limited access, tolerability concerns, and suboptimal efficacy in some populations underscore the need for novel pharmacotherapies targeting the underlying neurobiology of addiction.

In this context, the gut–brain axis (GBA) has emerged as a promising framework [6]. This bidirectional communication system involves neural, endocrine, and immune pathways linking the gastrointestinal tract and central nervous system (CNS) [7]. Among the hormones implicated in this axis, glucagon-like peptide 1 (GLP-1) has gained attention due to its dual metabolic and neuroregulatory actions. GLP-1 is secreted by intestinal L cells and brainstem neurons and exerts effects via GLP-1 receptors (GLP-1Rs), which are expressed in mesolimbic reward areas, including the ventral tegmental area (VTA), nucleus accumbens (NAc), amygdala, and hippocampus [7].

Building upon this foundation, the present review explores the therapeutic relevance of GLP-1 receptor agonists (GLP-1RAs) as neurometabolic modulators in the treatment of SUDs. By examining how these agents interact with key nodes of the reward circuitry—particularly within the mesocorticolimbic dopamine system—we aim to elucidate the mechanisms through which GLP-1 signaling may attenuate drug-seeking behavior, reduce relapse vulnerability, and modulate craving. In doing so, we contextualize these findings within broader frameworks of translational neuropsychopharmacology and systems biology. Ultimately, this review advocates for a paradigm shift in addiction medicine: one that recognizes the GBA not merely as a metabolic regulator, but as a critical entry point for future neurotherapeutic interventions.

## 2. The GLP-1 System: Physiology, Receptors, and CNS Distribution

### 2.1. GLP-1 Synthesis and Secretion

GLP-1 is a 30- to 31-amino-acid peptide hormone derived from the tissue-specific post-translational processing of proglucagon, a 160-amino-acid precursor encoded by the GCG gene located on chromosome 2q24.2. In intestinal L-cells, proglucagon is cleaved by prohormone convertase 1/3 (PC1/3) to generate GLP-1, along with other peptides such as GLP-2, oxyntomodulin, and peptide YY. These L cells are predominantly located in the distal small intestine and colon [8].

GLP-1 secretion is primarily stimulated by nutrient ingestion, exhibiting a biphasic release pattern: an initial rapid rise within 15–30 min post-meal, followed by a second minor peak at 90–120 min. The early phase is thought to be mediated by neuroendocrine pathways, including vagal afferents and enteric neurotransmitters such as acetylcholine and gastrin-releasing peptide. The later phase results from direct interaction of nutrients, particularly fats and carbohydrates, with L cells in the distal gut [9,10]. Recent studies have identified the mechanosensitive ion channel Piezo1 in intestinal L cells, suggesting that mechanical stretching of the intestinal wall can enhance GLP-1 secretion. Activation of Piezo1 leads to calcium influx and subsequent activation of the CaMKKβ/CaMKIV-mTORC1 signaling pathway, promoting GLP-1 production [11].

In addition to peripheral sources, GLP-1 is also synthesized in the CNS by preproglucagon-expressing neurons in the nucleus of the solitary tract (NTS) [12]. These neurons project to various brain regions involved in energy balance and reward, including the hypothalamus and VTA. Central GLP-1 plays a crucial role in regulating appetite, stress responses, and glucose homeostasis [13,14].

These insights into the spatiotemporal dynamics of GLP-1 synthesis and release underscore its multifaceted role as both a peripheral incretin and a central neuropeptide. The capacity of GLP-1 to influence neural circuits implicated in energy balance, stress responsiveness, and motivational drive is mediated through its interaction with GLP-1Rs, whose distribution and signaling properties within the CNS warrant detailed examination.

### 2.2. GLP-1Rs: Molecular Architecture, Central Distribution, and Intracellular Signaling

The GLP-1R is a class B G protein-coupled receptor (GPCR) that mediates the diverse physiological effects of GLP-1 in both peripheral and central tissues [15]. Structurally, GLP-1R comprises a large extracellular N-terminal domain responsible for ligand binding, seven transmembrane α-helices, and an intracellular C-terminal domain that interacts with G proteins and other signaling molecules [15]. Upon GLP-1 binding, GLP-1R undergoes conformational changes that activate intracellular signaling cascades, primarily through coupling with the stimulatory G protein (Gs), leading to the activation of adenylate cyclase and subsequent elevation in cyclic adenosine monophosphate (cAMP) levels [15]. This increase in cAMP activates downstream effectors such as protein kinase A (PKA) and exchange protein directly activated by cAMP 2 (Epac2), which modulate various cellular responses [8].

In the CNS, GLP-1R is widely expressed across multiple regions implicated in energy homeostasis, reward processing, and autonomic control [16]. Notably, high densities of GLP-1R are found in the hypothalamic nuclei, including the arcuate nucleus (ArcN), paraventricular nucleus (PVN), and dorsomedial hypothalamus (DMH), which are critical for regulating appetite and energy expenditure [17]. Additionally, GLP-1R is present in the nucleus tractus solitarius (NTS) and area postrema (AP) of the brainstem, regions involved in satiety signaling and autonomic regulation [10]. Importantly, GLP-1R is also expressed in mesolimbic structures such as the VTA and NAc, suggesting a role in modulating reward-related behaviors [14].

The activation of GLP-1R in these central regions influences various physiological and behavioral processes [8]. In the hypothalamus, GLP-1R activation suppresses food intake and promotes energy expenditure, contributing to body weight regulation. In the brainstem, GLP-1R signaling modulates gastric emptying and cardiovascular function [18]. In the mesolimbic system, GLP-1R activation has been shown to attenuate the rewarding effects of palatable food and addictive substances, indicating potential therapeutic applications for GLP-1R agonists in treating obesity and SUDs [19,20,21].

At the molecular level, GLP-1R activation leads to the stimulation of adenylate cyclase, resulting in increased cAMP production. The elevated cAMP levels activate PKA and Epac2, which in turn phosphorylate various target proteins, leading to alterations in gene expression, neurotransmitter release, and neuronal excitability. These signaling pathways are crucial for mediating the anorectic and neuroprotective effects of GLP-1R activation [22,23].

Taken together, the structural features and intracellular signaling pathways of GLP-1R provide the molecular substrate through which GLP-1 exerts its diverse central effects. However, it is the topographical distribution of these receptors across functionally distinct brain regions—particularly those implicated in motivation, reinforcement, and reward—that has garnered increasing attention in the context of addiction neurobiology. The following section examines in detail the localization of GLP-1Rs within key mesolimbic and corticolimbic nodes, with a focus on their functional relevance for neurobehavioral regulation.

### 2.3. Central Distribution of GLP-1Rs in Reward-Related Brain Regions

As mentioned, the GLP-1R is expressed in various regions of the CNS, including areas implicated in reward processing and addiction. Notably, GLP-1R expression has been identified in the VTA, NAc, amygdala, and hippocampus [24].

In the VTA, GLP-1R expression is present, but relatively sparse. Studies have shown that GLP-1R activation in the VTA can modulate dopaminergic activity, influencing reward-related behaviors. For instance, GLP-1R activation in the VTA has been associated with reduced intake of palatable foods and attenuated responses to addictive substances [25]. The NAc, a critical component of the mesolimbic reward pathway, also exhibits GLP-1R expression [26]. Activation of GLP-1R in the NAc has been linked to decreased motivation for rewarding stimuli, suggesting a role in modulating reward-seeking behavior [27]. In the amygdala, GLP-1R expression has been observed, particularly in the central and basolateral nuclei. GLP-1R activation in the amygdala may influence emotional aspects of reward processing and stress-related behaviors [28]. The hippocampus, involved in learning and memory, also expresses GLP-1R. GLP-1R activation in the hippocampus has been associated with neuroprotective effects and modulation of cognitive functions, which may indirectly affect reward-related behaviors [29].

Overall, the presence of GLP-1R in these reward-related brain regions suggests that GLP-1 signaling may play a significant role in modulating reward processing and addictive behaviors [6]. Further research is needed to elucidate the precise mechanisms by which GLP-1R activation influences these complex neural circuits.

### 2.4. Cross Talk Between Metabolic and Reward Signaling

The GLP-1 system plays a pivotal role in integrating metabolic and reward-related signaling pathways within the CNS. Beyond its established functions in glucose homeostasis and appetite regulation, GLP-1R activation has been implicated in modulating neurobiological mechanisms underlying addictive behaviors.

Preclinical studies have demonstrated that GLP-1R agonists, such as exendin 4 (Ex4) and liraglutide, attenuate drug-induced dopamine release in the NAc, a key region involved in reward processing. For instance, Ex4 administration has been shown to reduce cocaine-induced elevations in extracellular dopamine levels in the NAc, suggesting a dampening effect on the mesolimbic dopamine system [30]. Similarly, liraglutide has been reported to decrease alcohol-induced dopamine release in the NAc, further supporting the role of GLP-1R activation in modulating dopaminergic neurotransmission associated with substance use [31]. In addition to its effects on dopamine, GLP-1R activation influences other neurotransmitter systems implicated in reward and addiction. Notably, GLP-1R agonists have been found to modulate gamma-aminobutyric acid (GABA) signaling. Semaglutide, a long-acting GLP-1 analogue, has been shown to reduce alcohol consumption in rodents, an effect associated with alterations in central GABA neurotransmission [32]. Furthermore, GLP-1R activation has been reported to suppress GABA_A receptor-mediated currents in retinal ganglion cells, indicating a broader role in modulating inhibitory neurotransmission [33]. GLP-1R activation also affects glutamatergic signaling. In cerebellar slices, GLP-1 has been shown to enhance glutamate release at parallel fiber–Purkinje cell synapses via a presynaptic PKA signaling pathway, leading to increased excitatory synaptic transmission. This modulation of excitatory neurotransmission may contribute to the observed effects of GLP-1R agonists on reward-related behaviors [34].

Beyond the modulation of reward and metabolic circuits, emerging evidence suggests that GLP-1 receptor signaling may also influence central pathways involved in respiratory control and sleep regulation. GLP-1 receptors are expressed in brainstem regions implicated in respiratory rhythmogenesis, and their activation has been shown to stabilize breathing patterns and enhance respiratory drive in preclinical models [35]. These effects may be particularly relevant in patients with comorbid obesity and obstructive sleep apnea (OSA), conditions frequently co-occurring with SUDs such as alcoholism and opioid dependence [36,37]. Additionally, the bidirectional relationship between sleep disturbances and addiction has been increasingly recognized: substance use disrupts sleep architecture, while sleep disorders themselves may predispose to relapse and worsen addiction outcomes [38]. Collectively, these findings highlight the broader neuromodulatory role of GLP-1R agonists and underscore the need to explore their potential in treating sleep-related impairments in addiction medicine.

The GLP-1 system has emerged as a key integrative node linking metabolic regulation and neurobiological mechanisms of reward. GLP-1 receptors (GLP-1Rs), beyond their classical roles in glycemic control and satiety, are expressed in central regions associated with reward valuation, including the VTA, NAc, and prefrontal cortex. Activation of these receptors modulates mesocorticolimbic neurotransmission through dopaminergic, GABAergic, and glutamatergic pathways, which are critically involved in addictive behaviors. To provide a structured understanding of this neurometabolic interface, in the next section, we summarize preclinical findings on the effects of GLP-1R activation across different substance use models.

**Figure 1:**
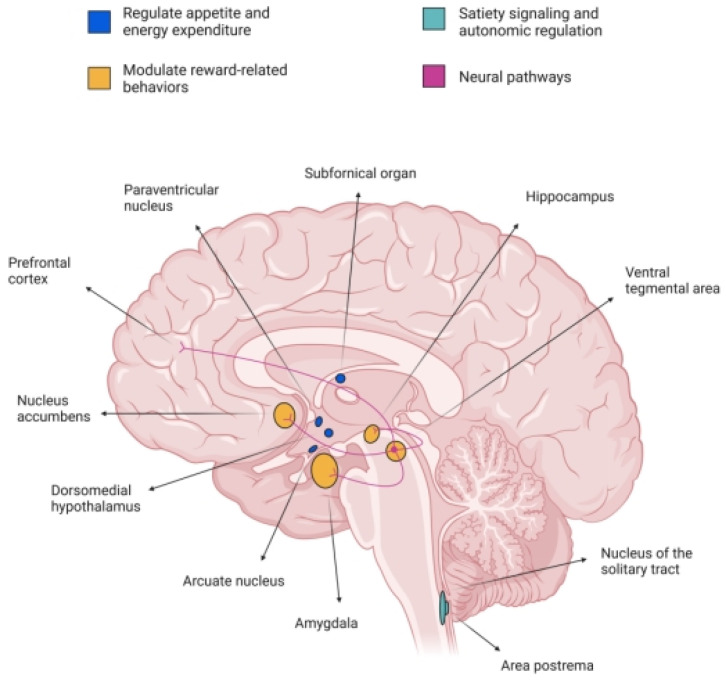
Interaction between GLP-1 signaling and neural reward pathways. GLP-1 receptors are expressed in multiple brain regions involved in appetite regulation, energy balance, autonomic control, and reward processing. The figure highlights key neuroanatomical sites—such as the arcuate nucleus, paraventricular nucleus, nucleus accumbens, amygdala, and ventral tegmental area—where GLP-1 signaling modulates both homeostatic and hedonic pathways. Colored nodes indicate functional roles: blue metabolic regulation, orange reward modulation, teal autonomic/satiety signaling, and purple interconnecting neural pathways.

Figure 1 presents the neurobiological interface between GLP-1 and reward-related signaling mechanisms.

### 2.5. Modulation of “Liking” and “Wanting” by GLP-1RAs: An Incentive-Sensitization Theory Perspective

Current findings, consistent with Berridge and Robinson’s incentive-sensitization theory, suggest that GLP-1RAs differentially impact two key aspects of addictive substance consumption: “liking” (hedonic pleasure) and “wanting” (motivational drive). This modulation appears to vary based on the specific substance, interindividual variability, and experimental paradigm [39].

Some studies have demonstrated that GLP-1 receptor activation decreases conditioned place preference (CPP) for alcohol, nicotine, cocaine, and amphetamines [40]. Since CPP is a behavioral marker of a substance’s hedonic value, these results indicate that GLP-1RAs may attenuate the affective component of reinforcement, i.e., “liking”. This aligns with Berridge and Robinson’s proposal that “liking” is a distinct hedonic process, separate from the dopaminergic system [41].

Conversely, research utilizing operant self-administration models shows that GLP-1RAs reduce the motivation to seek and consume drugs. This is evidenced by a decrease in the effort animals are willing to exert to obtain the substance [40,42,43]. These findings suggest that these compounds diminish the incentive salience of drug-related cues, affecting the “wanting” component without necessarily eliminating direct pleasure. Incentive-sensitization theory postulates that this “wanting” becomes hypersensitized in addiction, generating compulsive craving even without an increase in pleasure [39].

Crucially, some studies have revealed that GLP-1RAs can reduce drug-induced “wanting” without inducing aversion or interfering with natural rewards, such as the enjoyment of sweet foods. For instance, Ex4 can decrease alcohol self-administration without generating conditioned taste aversion, indicating that these medications selectively modulate addictive motivation while preserving natural hedonic pathways [41].

These actions are mediated by GLP-1 receptors located in key mesolimbic brain regions, including the VTA and the NAc, both of which are central to “wanting” as per Berridge and Robinson. In specific addiction models, additional neuronal pathways have been identified, such as the NTS–medial habenula (MHb)–interpeduncular nucleus (IPN) axis for nicotine and laterodorsal tegmental nucleus (LDTg)/VTA GABAergic projections for cocaine and alcohol [32]. These distinct neural pathways may differentially influence “liking” and “wanting” circuits, opening new avenues of research to understand how GLP-1RAs can treat pathological motivation without suppressing functional pleasure [41].

Importantly, the modulation of addictive behaviors by GLP-1RAs appears to differ across the distinct phases of the addiction cycle, engaging partially overlapping, but functionally divergent neurobiological substrates. During the intoxication phase, GLP-1R activation primarily targets mesolimbic dopaminergic circuits—including the VTA and NAc—to suppress drug-induced reinforcement and hedonic valuation, reflected behaviorally by reductions in conditioned place preference and self-administration. In contrast, during the withdrawal phase, GLP-1RAs appear to exert effects through broader circuits involving the lateral septum, hypothalamus, and brainstem nuclei, modulating stress responsivity, negative affect, and somatic symptoms, as evidenced in models of opioid and nicotine withdrawal. These regionally specific actions suggest that the neurobiological mechanisms underlying GLP-1–mediated attenuation of substance use behaviors are phase-dependent, with dopaminergic modulation predominating in intoxication and neuroendocrine or affective circuits contributing more prominently during withdrawal. This conceptual dissociation reinforces the therapeutic potential of GLP-1RAs to target multiple domains of addiction pathology through mechanistically distinct, yet complementary pathways.

**Figure 2:**
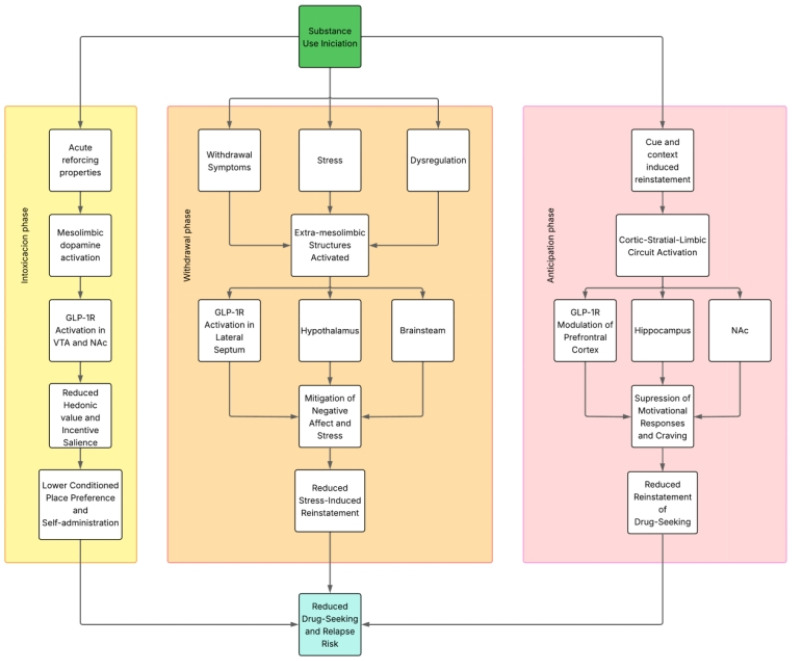
Potential involvement of GLP-1RAs across the phases of SUDs. This figure illustrates the potential involvement of GLP-1RAs in modulating neurobiological processes during the intoxication, withdrawal, and anticipation phases of SUDs. During intoxication, GLP-1R activation in the ventral tegmental area (VTA) and nucleus accumbens (NAc) reduces the hedonic and reinforcing properties of substances, leading to decreased conditioned place preference and self-administration. In the withdrawal phase, GLP-1RAs act on the lateral septum, hypothalamus, and brainstem to mitigate negative affect and stress, ultimately reducing stress-induced reinstatement. In the anticipation phase, modulation of prefrontal cortical regions and downstream targets such as the hippocampus and NAc contributes to decreased cue-induced reinstatement and craving. These distributed actions may collectively lower drug-seeking behavior and relapse risk, supporting the therapeutic potential of GLP-1RAs in addiction.

Considering the previously discussed information, GLP-1 analogues, by interacting with key structures within the reward circuitry—such as the mesolimbic and corticolimbic–striatal systems—may influence the distinct phases of the addiction cycle, including intoxication, withdrawal, and anticipation/craving [41]. Figure 2 illustrates these interactions.

## 3. Preclinical Insights into GLP-1 Receptor Modulation of Addictive Behaviors

Building on the mechanistic insights discussed above, this section reviews preclinical evidence supporting the role of GLP-1 receptor agonists in modulating addiction-related behaviors through central pathways. Beyond their metabolic effects, GLP-1RAs act on mesolimbic, hypothalamic, and brainstem circuits to influence reinforcement, craving, withdrawal, and relapse. These effects vary depending on the pharmacological profile of each substance and the neural systems involved. Accordingly, we present a substance-specific synthesis of experimental findings across alcohol, nicotine, psychostimulants, and opioids, highlighting both shared and distinct mechanisms underlying GLP-1RA efficacy in addiction models.

### 3.1. Preclinical Models of AUD

A growing body of evidence indicates that GLP-1RAs modulate alcohol-related behaviors via central mechanisms, particularly within mesolimbic circuits. Shirazi et al. (2013) showed that systemic and intra-VTA administration of GLP-1 or Ex4 reduced ethanol consumption in male Wistar rats without affecting food or water intake. These effects were more pronounced in high-alcohol-consuming animals, and microinjection into the VTA confirmed a critical role of mesolimbic GLP-1Rs. In parallel, GLP-1 suppressed ethanol-induced CPP in NMRI mice and blockade with exendin 9–39 increased drinking, supporting a role for endogenous GLP-1 signaling in the regulation of alcohol intake [44].

Egecioglu et al. (2013) further demonstrated that Ex4 reduced ethanol-induced hyperlocomotion and abolished the rise in NAc dopamine in mice. In both mice and rats, Ex4 disrupted CPP acquisition and expression and reduced voluntary alcohol intake. Notably, the study also employed a progressive ratio (PR) schedule, an operant conditioning paradigm in which the response requirement to obtain a reinforcer (e.g., a drug infusion) increases progressively following each successful trial. This schedule is commonly used to assess the motivational strength or *breakpoint*—the highest ratio completed—serving as a quantitative index of the subject’s willingness to work for the drug. Under this schedule, Ex4 significantly reduced the number of active lever presses and the breakpoint for ethanol without affecting inactive responses, indicating a selective reduction in the motivation to obtain alcohol [45].

Extending this work, Vallöf et al. (2019) used intra-NTS microinfusions of Ex4 to show that GLP-1R activation in this brainstem region attenuated alcohol-induced locomotor activity, NAc dopamine release, CPP, and ethanol intake. Blockade of NTS GLP-1Rs abolished the behavioral effects of systemic Ex4, identifying this region as both necessary and sufficient for mediating its anti-addictive actions [46].

In a related study, Vallöf et al. (2019) dissected the contribution of GLP-1Rs in the NAc shell (NAcS), anterior VTA (aVTA), posterior VTA (pVTA), and LDTg. Intra-NAcS and LDTg Ex4 infusions robustly reduced ethanol-induced locomotion and reward memory, while aVTA and pVTA showed limited or partial responsiveness. In high-alcohol-consuming rats, Ex4 microinfusions into NAcS and LDTg decreased ethanol intake without affecting general behavior. Moreover, GLP-1R expression in the NAc correlated positively with alcohol intake, suggesting adaptive upregulation of this signaling pathway in heavy drinkers [47].

Colvin et al. (2020) expanded the anatomical map of GLP-1–sensitive regions by showing that Ex4 microinjections into the VTA, NAc core and shell, lateral hypothalamus (LH), and dorsomedial hippocampus (DMHipp) suppressed alcohol intake. These effects were not observed in ArcN, PVN, or basolateral amygdala (BLA). In operant tasks for palatable food, Ex4 modulated both hypothalamic and mesolimbic targets, but only mesolimbic and hippocampal regions influenced alcohol-directed behaviors. This suggests a functional dissociation between GLP-1 modulation of metabolic and drug-related appetitive processes [48].

More recently, Aranäs et al. (2023) reported that semaglutide reduced both baseline and relapse-like alcohol consumption in rats, blunted ethanol-induced hyperlocomotion and NAc dopamine release, and increased expression of dopamine-metabolizing enzymes. Semaglutide was detected in the NAc following systemic injection, confirming central penetrance. Interestingly, while it did not affect alcohol-induced CPP, it selectively suppressed hedonic feeding and enhanced novelty-seeking behavior, consistent with broader mesolimbic modulation [49].

Taken together, these studies show that GLP-1RAs attenuate multiple domains of alcohol-related behavior—consumption, motivation, reward learning, and dopaminergic activation—through actions on key regions such as the VTA, NAcS, LDTg, LH, DMHipp, and NTS. These effects are dose-dependent, behaviorally specific, and anatomically localized, supporting the potential of GLP-1RAs as neurometabolic regulators of alcohol reinforcement and seeking. Their safety profile and CNS activity position them as promising candidates for the treatment of AUD, particularly in individuals with metabolic vulnerabilities.

### 3.2. Preclinical Models of Nicotine Use Disorder

Accumulating evidence suggests that the GLP-1 system modulates nicotine-related behaviors through central mechanisms that extend beyond traditional mesolimbic reward circuitry. The following studies systematically examine the role of GLP-1RAs—primarily Ex4 and liraglutide—in regulating nicotine intake-, reinforcement-, and withdrawal-associated phenotypes across a variety of rodent models.

Egecioglu et al. (2013) conducted a foundational study to investigate the impact of GLP-1R activation on nicotine-induced behavioral and neurochemical outcomes in male NMRI mice. Using a systemic administration protocol, mice received Ex4 (2.4 µg/kg, intraperitoneally) prior to exposure to nicotine (0.5 mg/kg, i.p.). Ex4 significantly attenuated nicotine-induced locomotor stimulation, as measured by open field activity, without affecting baseline locomotion. In vivo microdialysis of the NAc revealed that Ex4 blocked nicotine-evoked increases in extracellular dopamine concentrations. Importantly, these effects were not observed when Ex4 was administered alone, indicating a stimulus-dependent neuromodulatory profile. CPP testing demonstrated that a single administration of Ex4 was sufficient to abolish the expression of nicotine-induced CPP. Furthermore, repeated nicotine exposure under a sensitization protocol showed that Ex4 prevented the development of locomotor sensitization. These findings collectively support the hypothesis that GLP-1R activation dampens nicotine reward by reducing mesolimbic dopaminergic activation. The authors emphasized that Ex4 did not impair general activity or induce aversive effects, further validating the specificity of its action on nicotine reinforcement mechanisms [40].

Tuesta et al. (2017) expanded upon these pharmacological observations by identifying a discrete neuroanatomical circuit underlying GLP-1–mediated regulation of nicotine intake. Using a combination of chemogenetic, optogenetic, and pharmacological methods in wild-type and Glp-1R–deficient mice, the authors delineated an NTS → MHb → IPN pathway responsible for nicotine avoidance. They demonstrated that systemic nicotine activates GLP-1–producing neurons in the nucleus of the solitary tract (NTS), which in turn send excitatory projections to the MHb and IPN—regions classically involved in aversion. Ex4 and the DPP-4 inhibitor sitagliptin (which increases endogenous GLP-1 levels) both significantly reduced nicotine consumption in oral self-administration assays. Conversely, *GLP-1R* knockout mice consumed significantly more nicotine than wild-type controls. Site-specific knockdown of GLP-1R in the MHb or pharmacological antagonism of GLP-1Rs in the IPN increased nicotine intake and restored preference for nicotine-paired environments. Furthermore, optogenetic stimulation of GLP-1 projections to the MHb–IPN pathway abolished nicotine reward and suppressed intake without altering food consumption or inducing taste aversion. The data indicate that GLP-1 signaling through the MHb–IPN circuit suppresses nicotine reinforcement by promoting early avoidance responses, likely functioning as a homeostatic mechanism to prevent overconsumption. This study provided a mechanistic framework linking the GBA to habenular aversion circuits in the regulation of nicotine behavior [50].

Herman et al. (2023) evaluated the efficacy of liraglutide, a long-acting GLP-1R agonist, in modulating nicotine self-administration, relapse-like behavior, and withdrawal-associated hyperphagia in both male and female rats. Animals were trained to self-administer intravenous nicotine (0.03 mg/kg/infusion) over a 21-day period. Following the acquisition phase, liraglutide (25 µg/kg, i.p., daily) was administered beginning on the final day of self-administration and continued through extinction and reinstatement testing. Liraglutide significantly reduced nicotine-seeking behavior during cue- and nicotine-induced reinstatement sessions, suggesting attenuation of both drug-primed and conditioned stimulus-driven relapse vulnerability. Importantly, liraglutide also normalized high-fat diet (HFD) intake during withdrawal, a commonly observed phenotype in nicotine-abstinent individuals and a major contributor to smoking relapse in humans. The effect was observed in both sexes, although with some sex-specific variability in the magnitude and timing of response. Liraglutide administration did not reduce general food intake during baseline or produce motor suppression, highlighting behavioral specificity. This study is notable for integrating behavioral models of both addiction and metabolic dysregulation, reinforcing the dual utility of GLP-1R agonists in targeting comorbid risk factors associated with nicotine use disorder [51].

Collectively, the findings from these preclinical studies provide compelling evidence that GLP-1RAs modulate nicotine-related behaviors through both mesolimbic and habenula–brainstem pathways. Systemic and region-specific administration of GLP-1R agonists such as Ex4 and liraglutide reliably attenuate nicotine-induced locomotor stimulation, CPP, self-administration, and relapse-like behaviors without inducing malaise or generalized behavioral suppression. At the neurochemical level, GLP-1R activation consistently blunts nicotine-evoked dopamine release in the NAc, suggesting a reduction in the reinforcing efficacy of nicotine via modulation of dopaminergic transmission. Notably, beyond the canonical mesolimbic reward system, recent work has identified a critical role for GLP-1 signaling in the MHb and IPN. Activation of the NTS → MHb → IPN circuit by GLP-1 or its analogues suppresses nicotine intake and abolishes reward-related responses, while genetic or pharmacological disruption of this pathway enhances consumption. These findings suggest that GLP-1R activation functions not only to suppress reward salience but also to engage avoidance mechanisms that limit nicotine exposure. Moreover, GLP-1R agonists mitigate withdrawal-induced hyperphagia, a clinically relevant symptom that contributes to relapse during smoking cessation. The behavioral specificity, sex translatability, and anatomical precision observed across studies underscore the therapeutic potential of GLP-1–based interventions for nicotine use disorder. These results warrant further translational exploration of GLP-1R agonists, particularly in individuals for whom metabolic dysregulation and addiction vulnerability converge.

### 3.3. Preclinical Models of Cocaine and Psychostimulant Use Disorders

Cocaine and related psychostimulants such as amphetamines exert their addictive potential primarily via enhanced dopaminergic signaling in mesolimbic circuits, particularly through increased dopamine availability in the NAc. Traditional pharmacotherapies targeting monoamine systems have shown limited efficacy, prompting the investigation of alternative neuromodulatory systems such as the GLP-1 pathway. The following section summarizes key preclinical findings that examine the role of GLP-1 receptor (GLP-1R) agonism—primarily with Ex4—in modulating cocaine- and amphetamine-related behaviors.

Erreger et al. (2012) provided some of the earliest evidence that systemic GLP-1R activation modulates psychostimulant-related behavior. In male Sprague Dawley rats, peripheral administration of Ex4 (30 µg/kg, i.p.) reduced spontaneous locomotor activity and significantly attenuated the locomotor-stimulant effects of d-amphetamine (1 mg/kg, i.p.). These effects were paralleled by electrophysiological data showing decreased firing rates of VTA dopamine neurons. Ex4 did not affect blood glucose levels at behaviorally active doses, ruling out hypoglycemia as a confounding factor [52].

Graham et al. (2012) explored whether Ex4 could attenuate the conditioned rewarding effects of cocaine in mice. Using a cocaine-induced CPP paradigm, male C57BL/6J mice received Ex4 (10 µg/kg, i.p.) before the post-conditioning test session. Ex4 significantly reduced the expression of cocaine-induced CPP without affecting locomotor activity or inducing conditioned place aversion. Furthermore, Ex4 did not disrupt preference for natural rewards [53].

Egecioglu et al. (2013) expanded this evidence base by systematically evaluating the effects of Ex4 on locomotor activity, NAc dopamine release, and reward-associated learning induced by both cocaine and amphetamine. Using NMRI mice, systemic Ex4 (2.4 µg/kg, i.p.) attenuated hyperlocomotion induced by cocaine (15 mg/kg, i.p.) and amphetamine (2 mg/kg, i.p.) and abolished drug-evoked increases in extracellular dopamine in the NAc, as assessed via in vivo microdialysis. In parallel, Ex4 blocked cocaine-induced CPP and did not affect basal locomotion or food-related reward [40].

Harasta et al. (2015) investigated the contribution of GLP-1Rs within the lateral septum to cocaine-related behavior using a conditional knockout approach. Mice lacking GLP-1R selectively in the dorsal lateral septum exhibited enhanced cocaine-induced locomotor sensitization and increased CPP compared to wild-type controls. Restoration of GLP-1R expression specifically in the dorsal lateral septum of knockout mice normalized both phenotypes [54].

Reddy et al. (2016) explored molecular mechanisms downstream of GLP-1R activation in the lateral septum. Ex4 (2.4 µg/kg, i.p.) administration decreased cocaine-induced arachidonic acid signaling in the lateral septum and normalized cocaine-induced reductions in DAT function. These changes occurred in the absence of alterations in GLP-1R expression [55].

Schmidt et al. (2016) investigated the effects of the GLP-1R agonist Ex4 on cocaine reinforcement and relapse-like behavior using operant self-administration paradigms in male Sprague Dawley rats. Animals were trained to self-administer intravenous cocaine (0.75 mg/kg/infusion) under a fixed-ratio 1 schedule, followed by extinction and reinstatement testing. Acute systemic administration of Ex4 (2.4 µg/kg, i.p.) significantly reduced cocaine intake during maintenance sessions and attenuated both cue-induced and cocaine-primed reinstatement of drug-seeking behavior. Ex4 had no significant effects on inactive lever pressing or locomotor activity. Additionally, Ex4 increased c-Fos expression in the NAc [56].

Sirohi et al. (2016) evaluated the impact of the GLP-1R agonist liraglutide on the acute behavioral effects of cocaine in male Sprague Dawley rats. In a locomotor activity assay, systemic administration of liraglutide (100 or 200 µg/kg, s.c.) dose-dependently reduced cocaine-induced hyperlocomotion (10 mg/kg, i.p.) without altering baseline locomotion. In a separate experiment, liraglutide (200 µg/kg, s.c.) was administered prior to the post-conditioning test in a cocaine-CPP paradigm. Liraglutide abolished the expression of cocaine-induced CPP. Liraglutide did not induce conditioned taste aversion or suppress natural reward behaviors [57].

Sørensen et al. (2016) combined pharmacological and genetic approaches to assess the role of GLP-1R signaling in amphetamine-induced behavioral plasticity. Using both wild-type and *GLP-1R* knockout (*GLP-1R*^−/−^) mice, the authors tested the effects of Ex4 (2.4 µg/kg, i.p.) on the development and expression of amphetamine-induced CPP and locomotor sensitization. In wild-type mice, Ex4 significantly reduced both CPP expression and the progression of sensitization. *GLP-1R*^−/−^ mice exhibited enhanced responses to amphetamine and failed to respond to Ex4. Baseline locomotor activity was unaffected by Ex4 in either genotype [58].

Fortin and Roitman (2017) provided real-time neurophysiological evidence that GLP-1R activation disrupts phasic dopamine signaling associated with cocaine cues. Using fast-scan cyclic voltammetry in awake, behaving rats, the authors showed that ICV administration of Ex4 (0.3 µg) significantly reduced cue-induced dopamine transients in the NAc without affecting baseline dopamine levels. This blunting of cue-evoked dopamine signaling was temporally aligned with reductions in cue-elicited approach behavior [30].

Hernandez et al. (2018) further dissected the neuroanatomical basis for GLP-1R–mediated effects in cocaine seeking. Male Sprague Dawley rats were trained to self-administer cocaine and then tested in a reinstatement model after extinction. Microinjection of Ex4 into the VTA significantly attenuated cue-induced reinstatement of cocaine seeking. These effects were not associated with changes in general locomotion or inactive lever pressing. Systemic Ex4 mimicked the effects of local VTA infusion [59]. The same research group focused on the NAc as a downstream effector of GLP-1R signaling in cocaine-exposed rats. Repeated Ex4 administration during abstinence reduced cue- and drug-primed reinstatement of cocaine seeking. Electrophysiological recordings revealed that GLP-1R activation increased excitability of medium spiny neurons in the NAc, particularly D1-expressing subpopulations [27]. Subsequently, Hernandez et al. (2021) examined the contribution of the LDTg to GLP-1R–mediated suppression of cocaine seeking. Using optogenetics and pharmacological manipulations, the authors demonstrated that GLP-1R–expressing GABAergic neurons in the LDTg inhibit VTA dopamine neurons projecting to the NAc. Intra-LDTg infusion of Ex4 reduced cocaine seeking, and activation of LDTg → VTA GABAergic projections recapitulated these effects [60].

Merkel et al. (2025) integrated genetic, calcium photometry, and transcriptomic profiling to characterize a GABAergic NTS → VTA circuit mediating the behavioral effects of GLP-1R activation in cocaine models. Ex4 increased GCaMP6s signals in VTA-projecting NTS GABA neurons during cocaine-paired cue presentation, while chemogenetic silencing of this circuit abrogated the Ex4-induced reduction in cocaine seeking. Bulk RNA sequencing of NTS neurons identified enrichment of GLP-1R, Gad1, and neuropeptide Y transcripts [61].

The preclinical literature reviewed herein consistently demonstrates that activation of the GLP-1 receptor system modulates multiple domains of psychostimulant-induced behavior, including acute locomotor activation, conditioned reward, self-administration, and reinstatement. Across a range of rodent models and psychostimulants—namely cocaine and amphetamine—GLP-1R agonists such as Ex4 and liraglutide attenuate drug-induced hyperlocomotion, reduce CPP, and suppress mesolimbic dopamine signaling without impairing baseline locomotion or the valuation of natural rewards. These effects have been observed following both systemic administration and site-specific microinjections into key neuroanatomical targets, including VTA, NAc, lateral septum, and LDTg. Notably, mechanistic studies employing chemogenetics, fiber photometry, and genetic knockout models have identified distinct neural circuits through which GLP-1R agonists exert their effects. These include inhibitory GABAergic projections from the nucleus of the solitary tract (NTS) and LDTg to midbrain dopaminergic regions, as well as GLP-1R–dependent modulation of arachidonic acid signaling and DAT function in the septum. The specificity of these effects is further supported by the observation that GLP-1R agonists reduce reinstatement of cocaine-seeking behavior without altering inactive lever responses or general locomotor activity.

However, despite the consistency of these findings, several methodological limitations and sources of bias warrant consideration. First, most studies have been conducted in male rodents, limiting the generalizability of findings to female subjects and precluding analysis of sex-dependent responses. Second, while multiple brain regions have been explored in isolation, few studies have performed integrative circuit-level analyses across interconnected reward nodes, which may underestimate system-level interactions. Third, variability in dosing protocols, timing of administration (acute vs. repeated), and behavioral endpoints complicate direct comparisons between studies and hinder translational modeling. Fourth, some behavioral paradigms, such as CPP and locomotor sensitization, rely heavily on associative learning and may not fully capture compulsive aspects of drug seeking seen in clinical populations. Additionally, there is a relative paucity of long-term studies examining the persistence of GLP-1R–mediated effects beyond acute or subchronic treatment windows. Few studies address potential tolerance, compensatory neuroadaptations, or rebound effects following discontinuation. Moreover, while central mechanisms are implicated, only a subset of studies confirm BBB penetration, CNS bioavailability, or direct receptor engagement in target sites. Finally, most work to date has focused on Ex4 and liraglutide, and further investigation is needed to determine whether newer GLP-1R agonists with enhanced pharmacokinetic profiles (e.g., semaglutide) confer similar or superior efficacy in psychostimulant addiction models.

In conclusion, although preclinical evidence strongly supports the neuromodulatory role of GLP-1R signaling in reducing psychostimulant-induced behaviors, further research is required to refine our understanding of sex-specific effects, long-term efficacy, pharmacodynamic targets, and translational applicability. These efforts will be essential to advancing GLP-1–based therapies for cocaine and amphetamine use disorders.

### 3.4. Preclinical Models of Opioid Use Disorder

Opioid use disorder (OUD) remains one of the most devastating substance use pathologies worldwide, with high relapse rates and mortality. The reinforcing effects of opioids, such as heroin, morphine, and oxycodone, are largely mediated by mu-opioid receptor activation and downstream modulation of mesolimbic dopamine transmission. In recent years, preclinical studies have explored the potential of GLP-1R agonists to modulate opioid-induced behaviors by targeting overlapping circuits involved in reward and homeostasis. Below, we summarize key studies evaluating GLP-1R agonism in rodent models of opioid reinforcement, seeking, withdrawal, and relapse.

Douton et al. (2021) evaluated the effects of the GLP-1R agonist Ex4 (2.4 µg/kg) on heroin-seeking behavior in a rat model using a reward devaluation paradigm [62]. In this model, saccharin was paired with heroin availability, leading to avoidance of the cue due to its predictive value for drug access. Ex4 was administered during a 16-day abstinence period and again on the test day. Treatment reduced both cue-induced and heroin-induced reinstatement of drug seeking, although the latter effect was time-dependent and observed only when Ex4 was given 1 h (but not 6 h) prior to testing. Interestingly, while Ex4 did not alter saccharin intake during heroin access, a history of Ex4 treatment enhanced saccharin acceptance during extinction trials, suggesting mitigation of heroin-induced devaluation of natural reward. At the molecular level, Ex4 treatment increased orexin-1 receptor (OX1R) mRNA expression in the NAcS, a region implicated in drug motivation. These findings provide the first direct evidence that GLP-1R agonism attenuates both cue-driven and drug-induced reinstatement of heroin seeking in rats.

Zhang et al. (2020) assessed the role of GLP-1R activation in oxycodone self-administration and reinstatement [63]. In this study, male Sprague Dawley rats were trained to self-administer intravenous oxycodone under fixed ratio (FR) and PR schedules. An FR schedule is an operant reinforcement protocol where a specific, constant number of responses is required to obtain each unit of reinforcement. For example, under an FR schedule, each lever press results in a drug infusion. This paradigm allows for the evaluation of acquisition and maintenance of drug-seeking behavior. Systemic administration of Ex4 (2.4 µg/kg, i.p.) significantly decreased oxycodone intake under both schedules and reduced the motivation to obtain the drug, as indicated by a lower breakpoint in PR tests. Ex4 also suppressed cue-induced reinstatement of oxycodone seeking following extinction. Importantly, these effects were not accompanied by reductions in locomotor activity or food reinforcement, suggesting specificity for opioid-directed behavior. Immunohistochemical analysis revealed decreased c-Fos activation in the central amygdala and increased GLP-1R expression in the NAc, indicating recruitment of inhibitory control over affective and motivational drug circuits.

Łupina et al. (2020) investigated the impact of liraglutide on the affective and somatic components of opioid withdrawal in rats chronically exposed to morphine [64]. Male Wistar rats were administered escalating doses of morphine over 10 days to induce dependence, followed by withdrawal precipitated with naloxone (1 mg/kg, s.c.). Pretreatment with liraglutide (100 or 200 µg/kg, s.c.) significantly reduced somatic withdrawal signs such as wet-dog shakes, diarrhea, and teeth chattering. Moreover, liraglutide decreased anxiety-like behavior measured in the elevated plus maze and reversed withdrawal-induced reductions in sucrose preference, suggesting mitigation of negative affective states. Biochemical analysis showed that liraglutide normalized corticosterone levels and reduced activation of the hypothalamic–pituitary–adrenal (HPA) axis, providing a neuroendocrine correlate for its anxiolytic-like effects. These findings suggest that GLP-1R agonism attenuates both physical and emotional aspects of opioid withdrawal.

Bornebusch et al. (2019) investigated whether Ex4 could modulate the reinforcing or antinociceptive properties of morphine in mice [65]. Using male C57BL/6 mice, the authors evaluated the effects of Ex4 (2.4 µg/kg, i.p.) on morphine-induced CPP, locomotor sensitization, and analgesic tolerance. While Ex4 mildly reduced morphine-induced hyperlocomotion, it did not attenuate the expression of morphine-induced CPP, indicating a lack of effect on opioid reward in this model. Furthermore, Ex4 did not significantly prevent the development of morphine tolerance in antinociceptive assays (tail-flick and hot-plate tests). These findings suggest that under the conditions tested, Ex4 had limited efficacy in modulating morphine-induced reinforcement or tolerance, contrasting with results observed in models involving other drugs of abuse. The authors emphasize the need for further studies to clarify whether these results reflect a substance-specific effect or methodological boundaries.

Preclinical investigations into the effects of GLP-1RAs on opioid-related behaviors have yielded promising, but variable results. Several studies demonstrate that Ex4 and liraglutide reduce heroin- and oxycodone-seeking behavior, particularly under reinstatement paradigms triggered by drug-associated cues or priming. These effects have been observed without impairments in general locomotion or natural reward valuation and are accompanied by changes in molecular markers such as orexin receptor expression and limbic Fos activation. Additionally, GLP-1R activation has shown efficacy in mitigating withdrawal-related somatic signs and negative affect in morphine-dependent animals, potentially via HPA axis modulation.

However, not all findings align consistently. One study found that Ex4 did not reduce morphine-induced CPP or tolerance, suggesting that the efficacy of GLP-1R agonists may vary depending on the specific opioid, behavioral model, or species used. Furthermore, most data are derived from acute or subchronic interventions, and the long-term effects of GLP-1R agonism in the context of chronic opioid exposure remain largely uncharacterized.

Taken together, the available evidence supports the hypothesis that GLP-1R agonists may attenuate select components of opioid reinforcement, relapse, and withdrawal. Yet the translational potential of these findings requires further investigation, particularly regarding reproducibility, sex differences, and the differential pharmacological profiles of individual opioids.

Table 1 presents a summary of the most relevant preclinical studies evaluating the effects of GLP-1 analogues in the control of addiction.

To synthesize the findings discussed across different substance classes, Table 2 summarizes the neurobiological mechanisms by which GLP-1 receptor agonists modulate addiction-related behaviors in preclinical models. While mesolimbic dopamine suppression is a shared mechanism across alcohol, psychostimulant, and opioid use disorders, GLP-1R activation engages substance-specific circuits and neurotransmitter systems. These include modulation of orexin signaling and the HPA axis in opioid models, engagement of lateral septal and habenular circuits in psychostimulant models, and dopamine catabolism and hippocampal effects in alcohol paradigms. Such mechanistic differences may inform future efforts to personalize GLP-1–based interventions according to substance type and neurobiological profile.

## 4. Molecular and Pharmacokinetic Factors Shaping CNS Actions of GLP-1RAs

### 4.1. CNS Penetration of GLP-1RAs: Implications for Addiction

As previously reviewed, numerous preclinical studies have demonstrated that GLP-1RAs can modulate drug-related behaviors across a variety of addiction models, including those involving alcohol, nicotine, psychostimulants, and opioids. While these findings suggest a role for GLP-1 signaling in central reward pathways, a critical question remains regarding the extent to which pharmacological effects observed in the brain reflect direct engagement of central GLP-1 receptors versus indirect modulation through peripheral mechanisms. Given the peptide nature and structural diversity of GLP-1 analogues, their capacity to cross the BBB varies substantially and is governed by complex physicochemical and molecular factors. The following section critically examines current evidence concerning the CNS penetrance of GLP-1R agonists, exploring the relationship between their molecular properties and their ability to access brain tissue. This analysis is essential for interpreting preclinical results in neuropharmacological models and for assessing the translational potential of GLP-1–based therapies in the treatment of addiction.

The ability of GLP-1RAs to access the CNS has become a central consideration in understanding their potential role in modulating neuropsychiatric and reward-related processes, including addiction. Despite their origin as incretin-based therapies for type 2 diabetes and obesity, several GLP-1 analogues have demonstrated CNS effects that suggest, either directly or indirectly, interaction with brain targets. However, their capacity to cross the BBB varies substantially across compounds and is influenced by a combination of molecular weight, hydrophilicity, protein binding, susceptibility to enzymatic degradation, and structural modifications [66].

Peptides such as GLP-1 analogues are generally disadvantaged in terms of BBB penetration due to their relatively large size and low lipophilicity [67]. Passive diffusion across the BBB is significantly restricted for molecules exceeding ~500 Da, and most GLP-1 analogues greatly exceed this threshold. For instance, exenatide and lixisenatide have molecular weights of approximately 4200 and 4900 Da, respectively, while semaglutide and liraglutide range between 3700 and 4100 Da. Larger molecules like dulaglutide and albiglutide, which are fusion proteins with albumin or Fc domains, have molecular weights approaching or exceeding 60,000 Da, rendering them virtually impermeable to the BBB through classical routes. These size-related constraints are compounded by high hydrophilicity and a strong tendency toward plasma protein binding, particularly for semaglutide and liraglutide, whose fatty acid modifications promote albumin association and prolong plasma half-life, but may limit unbound drug fractions capable of CNS entry [68,69]. Table 3 summarizes the most important pharmacokinetic and physicochemical properties of GLP-1 analogues.

Nevertheless, preclinical and clinical data suggest that several GLP-1 analogues are able to exert central effects, and in some cases have been directly detected within brain tissue. Exenatide has been observed to accumulate in the brain parenchyma of rodent models, and lixisenatide has demonstrated relatively rapid CNS penetration, likely due to its smaller size, lack of extensive protein binding, and susceptibility to endocytosis [70]. Liraglutide has also been reported to cross the BBB to a limited extent, with pharmacologically relevant concentrations detected in discrete regions such as the hypothalamus and brainstem [66,70]. Semaglutide, while more lipophilic and longer-acting than other peptides in the class, appears to exhibit modest brain penetrance, possibly limited by its strong affinity for albumin and relatively high molecular weight [71]. Although dulaglutide is a high-molecular-weight fusion protein typically excluded from passive diffusion across the BBB, recent studies have demonstrated that it can reach multiple brain regions following intranasal administration, including the neocortex and hippocampus. While its CNS entry may be limited after systemic delivery, its ability to exert central effects—especially under specific delivery conditions—should not be dismissed. The evidence indicates that dulaglutide may modulate central neurocognitive pathways through both direct brain access and peripheral-to-central signaling mechanisms [70].

While nasal administration has been explored as a strategy to bypass the blood–brain barrier (BBB), the extent to which GLP-1 analogues benefit from this route depends heavily on their molecular characteristics. Peptides with high molecular weight, low lipophilicity, and strong plasma protein binding—such as most GLP-1 receptor agonists—are generally limited in their ability to diffuse across the BBB, even when administered nasally. Although some studies have demonstrated brain distribution following intranasal delivery for specific peptides, this cannot be generalized to all GLP-1 analogues. Therefore, the ability of GLP-1 receptor agonists to reach central targets via the nasal route should be interpreted cautiously and in direct relation to their physicochemical properties, including size, enzymatic stability, and affinity for transporter systems.

The mechanisms enabling CNS access for GLP-1 analogues are not fully understood, but several routes have been proposed [72]. One possibility is receptor-mediated or adsorptive transcytosis across brain endothelial cells, although direct evidence for this process is limited and likely molecule-specific. More plausibly, these agents may access the brain via regions lacking classical tight-junction architecture, such as the AP, subfornical organ, or median eminence—collectively known as circumventricular organs [67]. These structures allow for more permissive diffusion of blood-borne peptides and may serve as critical nodes for GLP-1 action [72]. Additionally, some CNS effects may be mediated peripherally through vagal afferent activation or endocrine-to-neural relay mechanisms that secondarily influence brain function without requiring parenchymal drug accumulation [8].

**Figure 3:**
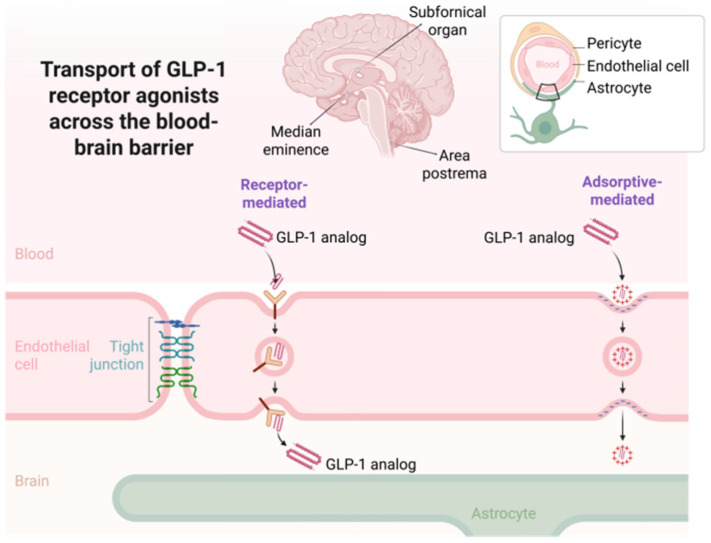
Putative mechanisms underlying CNS access of GLP-1 analogues. Although receptor-mediated or adsorptive transcytosis across the blood–brain barrier remains speculative and may be molecule-specific, GLP-1 analogues are thought to reach the brain primarily via circumventricular organs, such as the area postrema, subfornical organ, and median eminence. These regions lack classical tight-junctions and enable facilitated diffusion of circulating peptides, potentially mediating central GLP-1 receptor engagement. This figure is not drawn to scale and is intended solely to illustrate the conceptual relationship between the structures.

The extent to which these entry routes and physicochemical profiles translate into functional CNS engagement appears to be highly variable across molecules. While lixisenatide and exenatide demonstrate relatively rapid and direct action within central circuits, liraglutide and semaglutide may exert their effects more slowly or indirectly, potentially relying on longer systemic exposure, slow diffusion, or action through peripheral–central pathways. The lack of BBB penetration for dulaglutide and albiglutide, on the other hand, suggests that any CNS effects observed with these agents are likely secondary to peripheral metabolic or neurohumoral changes rather than direct receptor engagement in the brain. Figure 3 illustrates the main mechanisms by which GLP-1RAs access the CNS.

In conclusion, the degree to which GLP-1RAs cross the BBB is determined by a complex interplay of molecular and physicochemical factors. While smaller, unbound analogues with reduced protein affinity may access the CNS more readily, larger and highly bound compounds are effectively excluded. Despite these differences, several analogues can modulate brain-related functions, either through direct parenchymal access or via circumventricular and peripheral afferent mechanisms. Understanding these dynamics is critical for optimizing the use of GLP-1–based agents in disorders with central components, including neurodegeneration, psychiatric illness, and addiction. These insights also underscore the need for molecule-specific evaluation when interpreting or predicting CNS effects of this pharmacological class.

### 4.2. Pharmacokinetic Differences Between Endogenous GLP-1 and GLP-1 Receptor Agonists: Mechanisms and DPP-4 Resistance

As previously described, glucagon-like peptide 1 (GLP-1) is an endogenous incretin that regulates blood glucose levels through glucose-dependent stimulation of insulin secretion, inhibition of glucagon release, delayed gastric emptying, and appetite suppression. However, its direct therapeutic application is limited by its rapid inactivation via the enzyme dipeptidyl peptidase 4 (DPP-4), which cleaves GLP-1 (7–36) at position 8 to generate the inactive metabolite GLP-1 (9–36), resulting in a plasma half-life of only 1 to 2 min. The pharmacological development of GLP-1 receptor agonists (GLP-1RAs) has focused on introducing specific structural modifications to enhance resistance to DPP-4 degradation, extend half-life, and improve bioavailability. These agents exhibit partial or full sequence homology with human GLP-1, but incorporate strategic alterations—such as N-terminal protection or enhanced affinity for plasma proteins like albumin—as summarized in Table 4 [8].

All GLP-1RAs retain the capacity to activate the GLP-1 receptor (GLP-1R), thereby initiating intracellular signaling cascades—including cAMP accumulation, protein kinase A (PKA) activation, and PI3K/Akt pathway engagement—underlying their endocrine, metabolic, and neuroprotective effects [8].

A comprehensive comparison of the pharmacological and clinical characteristics of the major GLP-1RAs is provided in Table 3, encompassing parameters such as elimination, half-life, and adverse effect profiles. This information is critical for understanding the clinical implications of molecular design and for guiding the rational use of GLP-1RAs across diverse therapeutic contexts.

## 5. Clinical Evidence for GLP-1RAs in the Treatment of SUDs

### 5.1. Translational Perspectives on GLP-1R Modulation in AUD

A growing body of translational research highlights the therapeutic potential of GLP-1R agonists in AUD, bridging mechanistic findings from animal models with genetic and neurobiological data in humans. In a study by Suchankova et al. (2015), multiple lines of evidence converged to implicate GLP-1R signaling in the pathophysiology of AUD. Genetic association analyses in two independent cohorts identified the GLP-1R rs6923761 G > A (168Ser) variant as a significant predictor of AUD risk, particularly among male and nicotine-using subgroups. This variant was also associated with greater self-administered alcohol volume and higher peak breath alcohol concentrations during intravenous alcohol self-administration in healthy participants. Neuroimaging data further indicated that carriers of the 168Ser allele displayed enhanced blood oxygen level–dependent activation in the globus pallidus during feedback-related reward processing, suggesting altered reinforcement sensitivity within basal ganglia circuits [73].

In a complementary preclinical component of the same study, administration of the GLP-1R agonist AC3174 reduced ethanol intake in alcohol-dependent but not nondependent C57BL/6J mice exposed to a chronic intermittent ethanol vapor paradigm. This treatment effect persisted for at least five days following cessation of the drug, implying neuroadaptive changes in GLP-1–sensitive circuits following chronic alcohol exposure. These findings suggest that GLP-1R stimulation may exert differential effects depending on the neurobiological state associated with alcohol dependence [73].

Additional insights were provided by Farokhnia et al. (2022), who analyzed human laboratory and postmortem data to characterize the interaction between alcohol and the endogenous GLP-1 system. In four distinct experimental protocols, alcohol administration—via both oral and intravenous routes—produced significant reductions in peripheral active GLP-1 concentrations in individuals with AUD. This suppression was observed consistently across variable and fixed dosing paradigms. In postmortem brain tissue, GLP-1R mRNA expression in the hippocampus was significantly elevated in individuals with AUD compared to controls, with a similar trend observed in the prefrontal cortex. Exploratory correlation analyses revealed associations between hippocampal GLP-1R expression and measures of alcohol intake, while both hippocampal and prefrontal GLP-1R levels correlated with cigarette smoking behavior [74].

These observations support the existence of a bidirectional regulatory relationship between alcohol exposure and GLP-1 signaling at both peripheral and central levels. The consistent downregulation of peripheral GLP-1 following alcohol intake, coupled with upregulated central GLP-1R gene expression in AUD patients, suggests compensatory adaptations within the GLP-1 axis. Moreover, the convergence of genetic risk, hormone dynamics, and receptor expression reinforces the biological plausibility of targeting the GLP-1 system for therapeutic intervention. This body of evidence provides a mechanistic rationale for advancing GLP-1R agonists as candidate pharmacotherapies for AUD, with relevance for individuals characterized by specific genetic profiles, nicotine co-use, or metabolic vulnerability. Further investigation is warranted to elucidate the neuroendocrine mechanisms underlying these interactions and to identify patient subgroups most likely to benefit from GLP-1–based treatments.

### 5.2. Observational Studies on GLP-1R Agonists and Substance Use Outcomes

The clinical potential of GLP-1RAs in modulating substance use behaviors has been increasingly recognized through preclinical and early translational studies. In parallel, large-scale observational studies leveraging electronic health records (EHRs), self-report datasets, and real-world registries are beginning to characterize the association between GLP-1RA use and substance-related outcomes. The following section critically reviews four recent observational investigations that examined the relationships between GLP-1RA exposure—particularly semaglutide and tirzepatide—and various substance use metrics in humans.

Quddos et al. (2023) conducted a mixed-method investigation combining social media analytics and a remote self-report study in individuals with obesity (BMI ≥ 30) who were current alcohol consumers. Participants either self-administered semaglutide (GLP-1RA), tirzepatide (dual GLP-1/GIP RA), or were included in a control group with no relevant medication. Text mining of ~68,000 Reddit posts identified 1580 alcohol-related mentions, with 71% describing reduced cravings or desire to drink under GLP-1RA treatment. The remote study (*n* = 153) revealed significantly lower alcohol intake, binge drinking frequency, and AUDIT scores in medicated participants compared to baseline and controls. These results provide preliminary real-world evidence that GLP-1RAs may decrease alcohol consumption and subjective alcohol effects in people with obesity [75].

Tsermpini et al. (2022) performed a retrospective analysis using EHR data to examine the prevalence of AUD diagnoses in individuals prescribed GLP-1RAs for type 2 diabetes. Although the study did not focus on behavioral endpoints, they observed that patients with a documented history of AUD had lower odds of AUD recurrence during GLP-1RA treatment compared to those on other antidiabetic medications. These associations were independent of glycemic control and suggest a potential protective role of GLP-1 signaling in individuals with comorbid metabolic and alcohol-related disorders [76].

Wang et al. (2024) utilized the TriNetX research network, encompassing over 100 million patients, to conduct a retrospective cohort study assessing the impact of semaglutide on cannabis use disorder (CUD) in obese individuals. Although the focus was on cannabis rather than alcohol, the study demonstrated that semaglutide users had significantly lower incidence and recurrence of CUD compared to those receiving non-GLP-1–based anti-obesity treatments. The effect was consistent across sex, age, and race subgroups and was replicated in a secondary cohort with type 2 diabetes. These findings imply that GLP-1RAs may broadly attenuate addictive behaviors beyond alcohol and nicotine [77].

Qeadan et al. (2024) analyzed Medicaid data from multiple U.S. states to explore associations between GLP-1RA use and opioid-related outcomes. Patients prescribed semaglutide or liraglutide were less likely to receive opioid prescriptions or experience opioid-related hospitalizations during the observation period. These associations remained significant after adjusting for baseline pain diagnoses, comorbidities, and socioeconomic factors. While causality cannot be inferred from this study, the data align with a growing body of literature suggesting that GLP-1 signaling may influence motivational processes relevant to opioid use [78].

Collectively, these observational studies offer emerging evidence that GLP-1RAs may confer protective effects against a spectrum of SUDs, including alcohol, opioids, and cannabis. While confounding, selection bias, and self-report limitations remain inherent to such designs, the convergence of findings across diverse populations and analytic strategies strengthens the plausibility of a neurometabolic mechanism underlying these associations. Future research should prioritize prospective, controlled trials to validate these real-world findings and identify predictive markers of response in metabolically vulnerable individuals.

### 5.3. Randomized Controlled Trials Assessing GLP-1R Agonists in AUD

In recent years, the translational potential of GLP-1 receptor (GLP-1R) agonists for the treatment of AUD has been investigated in human clinical trials. Two randomized, placebo-controlled studies have evaluated the efficacy of GLP-1 analogues—exenatide and dulaglutide—on alcohol consumption outcomes in adult participants, offering distinct, but complementary perspectives on their utility in addiction medicine.

Klausen et al. (2022) conducted a 26-week double-blind, placebo-controlled trial in 127 treatment-seeking individuals with AUD to evaluate the effects of exenatide (2 mg subcutaneously, once weekly) as an adjunct to standard cognitive–behavioral therapy. The primary endpoint—reduction in the number of heavy drinking days—did not significantly differ between the treatment and placebo groups. However, exploratory analyses revealed a statistically significant reduction in heavy drinking days and total alcohol intake in the subgroup of patients with obesity (BMI > 30 kg/m^2^), suggesting a potential phenotype-specific treatment effect. Neuroimaging sub-studies provided additional mechanistic insights: functional MRI (fMRI) revealed a reduction in alcohol cue reactivity in the ventral striatum and septal area among patients receiving exenatide, while single-photon-emission computed tomography (SPECT) imaging showed decreased DAT availability in the striatum, caudate, and putamen. These neurobiological findings indicate central engagement of mesolimbic circuits relevant to addiction and support a potential role for GLP-1R agonists in modulating incentive salience and dopaminergic tone, even in the absence of overt clinical behavioral differences [79].

Probst et al. (2023) conducted a predefined secondary analysis of a 12-week randomized trial originally designed to evaluate the effects of dulaglutide (1.5 mg weekly) on smoking cessation. Among the 151 participants who reported alcohol consumption at baseline, dulaglutide significantly reduced alcohol intake compared to placebo, with a 29% relative reduction at week 12 (*p* = 0.04). This effect was further strengthened after adjustment for education (36% reduction; *p* = 0.004). Importantly, changes in alcohol consumption were independent of smoking cessation status, suggesting a direct effect of dulaglutide on alcohol intake. While the subgroup of heavy drinkers was too small for definitive conclusions, the findings echo those of Klausen et al. in suggesting that GLP-1R agonists may modulate alcohol-related behaviors in individuals not specifically selected for AUD. The predominantly obese profile of the study population (91% with BMI > 29.9 kg/m^2^) further aligns with the hypothesis that GLP-1 signaling may exert enhanced anti-reward effects in metabolically dysregulated individuals [80].

These trials highlight both the promise and complexity of GLP-1R agonists as potential pharmacotherapies for alcohol-related disorders. While primary endpoints of alcohol use reduction were not universally achieved, consistent neuroimaging evidence and subgroup-specific effects point to meaningful modulation of reward pathways. Future studies should aim to identify clinical and biological predictors of response, evaluate long-term efficacy and safety, and determine whether metabolic profiles, such as obesity or insulin resistance, influence the therapeutic impact of GLP-1–based interventions in AUD.

A recent systematic review by Subhani et al. (2024) synthesized clinical and observational data regarding the effects of GLP-1RAs on alcohol use. The review encompassed six studies—two randomized controlled trials and four observational investigations—totaling over 88,000 participants. Despite considerable methodological heterogeneity across studies, the collective findings suggest that GLP-1RAs may exert modulatory effects on alcohol-related behaviors, particularly in metabolically vulnerable populations.

While primary endpoints were not consistently met across trials, several studies reported reductions in alcohol intake within subgroups characterized by obesity or high baseline consumption. These effects were complemented by neuroimaging data demonstrating attenuated alcohol cue reactivity and reduced striatal DAT availability, indicating central engagement of reward-related pathways. Observational studies further support these associations, identifying reductions in alcohol use, binge drinking, and alcohol-related healthcare utilization among GLP-1RA users in real-world populations. However, these findings are constrained by the inherent limitations of non-randomized designs, including confounding and selection bias.

The reviewed evidence also supports the behavioral specificity of GLP-1RAs, as reductions in alcohol use were not accompanied by signs of generalized malaise or nonspecific behavioral suppression. Notably, most studies reported greater effects in individuals with co-occurring obesity, suggesting a phenotype-dependent interaction between metabolic status and reward modulation. The consistent tolerability profile across studies, with gastrointestinal symptoms being the most frequently reported adverse effects, reinforces the clinical feasibility of repurposing GLP-1RAs in this context.

Clinical trials investigating GLP-1 receptor agonists for reducing alcohol consumption have used established dosing regimens. In a study by Klausen et al., exenatide was administered at a dose of 2 mg subcutaneously once weekly for 26 weeks. In a study by Probst et al., dulaglutide was given at a dose of 1.5 mg subcutaneously once weekly for 12 weeks. Both agents were used in addition to standard behavioral interventions and followed administration protocols approved for their metabolic indications.

As discussed in recent pharmacoepidemiological literature (Echeverry-Guerrero et al., 2024), nausea, vomiting, and diarrhea may occur in up to 50% of users, especially during the early phases of treatment. These effects are typically dose-dependent and tend to diminish over time, but may contribute to treatment discontinuation in a subset of patients. More serious complications, such as gastroparesis and bowel obstruction, though less frequent, have been reported and warrant close monitoring, particularly in vulnerable populations [81].

In summary, although current clinical data remain preliminary, the convergence of behavioral, neurobiological, and epidemiological evidence indicates that GLP-1RAs may influence alcohol-related behaviors through mechanisms involving central reward circuitry. These findings justify continued investigation through prospective, adequately powered trials aimed at identifying the clinical phenotypes and neurobiological substrates most responsive to GLP-1–based interventions in AUD.

## 6. Translational Perspectives and Future Directions

The growing body of evidence reviewed throughout this article positions GLP-1RAs as promising pharmacological tools for the treatment of SUDs. However, the path from preclinical promise to clinical implementation is characterized by complex challenges that must be critically addressed to enable successful translation. This section outlines key translational considerations, emerging mechanistic insights, and strategic directions for future research.

One of the central considerations in the translational trajectory of GLP-1RAs is their CNS pharmacokinetics. While numerous preclinical studies demonstrate robust effects of GLP-1RAs on addiction-related behaviors through modulation of mesolimbic circuits, the assumption of direct CNS penetration has been increasingly questioned. Empirical data indicate that large, acylated GLP-1 analogues such as semaglutide and liraglutide exhibit poor permeability across the BBB under physiological conditions. Studies using radiolabeled and fluorescently tagged analogues in rodent models have shown that these compounds primarily accumulate in circumventricular organs (e.g., AP, median eminence), with minimal parenchymal distribution. Even short-acting compounds like Ex4 exhibit limited penetration, and their CNS effects may depend on indirect mechanisms such as vagal afferent activation or diffusion via tanycytic transport from cerebrospinal fluid. These findings emphasize the need to reconsider the CNS bioavailability of GLP-1RAs and to investigate alternative delivery strategies (e.g., intranasal administration) and molecular designs that improve brain penetrance.

Despite the pharmacokinetic limitations, the neuroanatomical distribution of GLP-1 receptors supports their mechanistic relevance in addiction. GLP-1Rs are expressed in key reward-processing structures, including the VTA, NAc, lateral septum, hippocampus, and MHb. Preclinical studies have demonstrated that activation of GLP-1Rs in these regions modulates DAT function, attenuates drug-induced dopamine release, and reduces reinstatement of drug-seeking behaviors. These neurochemical effects are consistent with clinical neuroimaging data showing reduced alcohol cue reactivity and striatal DAT availability in patients receiving exenatide. Nonetheless, additional studies are required to directly map CNS drug concentrations to functional outcomes in both animal and human models.

Another translational gap arises from the limited generalizability of preclinical models [82]. Most rodent studies use young adult males under highly controlled laboratory conditions. Such models do not adequately reflect the heterogeneity of human SUD populations, which differ in sex, age, comorbidities, and environmental context. For example, recent work has highlighted sex-based differences in the efficacy of GLP-1RAs, with male rodents showing greater reductions in ethanol intake and mesolimbic neurotransmitter activity compared to females. These differences may be driven by variations in GLP-1 receptor expression, estradiol signaling, or pharmacokinetics. Future preclinical studies should incorporate both sexes and model chronic, relapsing drug use to enhance ecological validity.

Pharmacogenetics represents another frontier in translational addiction research. Human studies have identified several polymorphisms in the *GLP-1R* gene that influence glycemic response to GLP-1RAs and may also modulate neurobehavioral outcomes. For instance, the rs6923761 (Gly168Ser) variant has been associated with altered alcohol self-administration and enhanced striatal activation during reward tasks [83]. Additional variants in *ARRB1* (e.g., *Thr370Met*) and other metabolic loci (e.g., *CTRB1*, *CHST3*) may further stratify individuals in terms of responsiveness, tolerance, and therapeutic outcomes [84]. These insights underscore the importance of integrating genomic profiling into future clinical trials to support personalized medicine approaches.

Beyond genetic variability, differences in metabolic phenotype may significantly influence treatment response. Observational and interventional studies have consistently shown stronger anti-addictive effects of GLP-1RAs in individuals with obesity or insulin resistance. These metabolic profiles may be associated with altered gut–brain signaling, differential receptor expression, or neuroinflammation, all of which can modulate reward processing. Future studies should evaluate GLP-1RA efficacy across stratified metabolic subgroups to determine optimal therapeutic windows and dosing strategies.

From a methodological standpoint, extrapolating from preclinical to clinical findings necessitates careful interpretation. Controlled environments in animal laboratories cannot recapitulate the complexity of real-world clinical settings. Discrepancies in species-specific drug metabolism, receptor distribution, and behavioral repertoire may lead to both false positives and negatives. Classical examples include thalidomide and aspirin, where toxicity profiles diverged significantly between rodents and humans. These limitations reinforce the need for translational biomarkers, such as neuroimaging, pharmacokinetic modeling, and neuroendocrine readouts, to bridge preclinical and clinical data.

Ethical considerations are equally crucial in the design of addiction trials involving GLP-1RAs. Individuals with SUDs often exhibit impaired decision-making capacity and are vulnerable to coercion. Clinical protocols must ensure rigorous informed consent, protect against undue inducement, and provide long-term follow-up. Trial designs should integrate multidisciplinary oversight, including addiction specialists, ethicists, and patient advocates. Additionally, ethical concerns arise regarding potential off-label use of GLP-1RAs in high-risk populations without sufficient safety data, particularly in individuals without comorbid metabolic disorders.

Despite encouraging preclinical results and early clinical signals, several limitations constrain the translational potential of GLP-1RAs for the treatment of SUDs. Most existing studies are preclinical or involve small, short-term clinical cohorts, limiting the robustness of efficacy and safety conclusions. Moreover, pharmacokinetic constraints—such as limited BBB permeability and variable CNS distribution—may restrict the central effects of certain GLP-1RAs. Gastrointestinal side effects, frequently observed across this drug class, pose additional challenges for adherence, particularly in populations with high vulnerability to treatment dropout. Importantly, treatment response appears to vary by metabolic status, with greater efficacy reported in individuals with obesity, suggesting the need for precision approaches. Finally, the neurobiological heterogeneity across SUDs underscores the importance of substance-specific clinical trials tailored to distinct mechanisms and outcome measures.

Looking ahead, several strategic directions can accelerate the clinical development of GLP-1RAs in addiction medicine. First, novel analogues with enhanced central penetration or receptor selectivity should be prioritized. Second, combination therapies integrating GLP-1RAs with dopaminergic, GABAergic, or neuroimmune modulators may yield synergistic effects. Third, the incorporation of digital phenotyping, neuroimaging, and multi-omics platforms will facilitate mechanistic understanding and patient stratification. Lastly, expanding clinical trials to include women, adolescents, and individuals with polysubstance use will enhance the generalizability and equity of future treatment strategies.

In conclusion, while substantial progress has been made in elucidating the role of GLP-1 signaling in addiction, significant translational hurdles remain. Bridging the gap from bench to bedside will require integrative, interdisciplinary approaches that account for molecular complexity, individual variability, and ethical responsibility. With continued refinement, GLP-1RAs may redefine the therapeutic landscape for SUDs, offering targeted, biologically informed interventions for populations historically underserved by existing treatments.

## 7. Conclusions

The emergence of GLP-1RAs as neurometabolic modulators represents a significant paradigm shift in the pharmacotherapy of SUDs. Initially developed for metabolic conditions such as type 2 diabetes and obesity, these agents have demonstrated the capacity to modulate reward-related behaviors across a diverse range of preclinical addiction models, including alcohol, nicotine, psychostimulants, and opioids. Mechanistic investigations have consistently implicated central GLP-1R signaling in mesocorticolimbic regions such as the VTA, NAc, lateral septum, and MHb, underscoring their relevance to the neurobiology of reinforcement, craving, and relapse.

Preclinical findings show remarkable consistency: GLP-1R agonists reduce voluntary substance intake, block drug-induced dopaminergic activation, inhibit conditioned reward learning, and attenuate relapse-like behaviors. These effects are observed across substances and models, including operant self-administration, CPP, reinstatement paradigms, and neurochemical readouts. Importantly, GLP-1RAs exert their behavioral effects without producing general malaise or impairments in natural reward valuation, highlighting their specificity and translational promise.

Clinical and observational data, while still limited in scope and heterogeneity, support the translational potential of these findings. Randomized controlled trials suggest that GLP-1RAs can modulate alcohol consumption and neural reward responses in select subgroups, particularly among individuals with obesity. Observational studies further reveal reductions in alcohol, opioid, and cannabis use in real-world populations treated with GLP-1RAs, reinforcing the clinical relevance of GBA signaling in addiction.

Nonetheless, significant challenges must be addressed to fully realize the therapeutic potential of GLP-1RAs in addiction medicine. Chief among these are limitations in BBB permeability, the reliance on male-dominant animal models, the absence of standardized behavioral endpoints, and the variability in CNS penetrance across GLP-1 analogues. Moreover, the influence of pharmacogenetic polymorphisms, metabolic phenotypes, and sex differences remains incompletely understood.

Future research must prioritize rigorous translational frameworks that integrate molecular, neurophysiological, and behavioral data across species. This includes the development of novel GLP-1RAs with optimized central bioavailability, the implementation of precision medicine approaches leveraging pharmacogenomic and metabolic profiling, and the design of ethically robust, adequately powered clinical trials involving diverse populations.

In summary, GLP-1RAs offer a biologically plausible, mechanistically grounded, and clinically actionable strategy for modulating addictive behaviors. While not a universal solution, their pleiotropic effects on central and peripheral pathways render them uniquely suited for addressing the complex interplay of metabolic and neuropsychiatric factors in SUDs. With continued refinement, GLP-1–based interventions may complement and enhance existing therapeutic paradigms, contributing to a more nuanced and individualized approach to addiction treatment.

## Figures and Tables

**Table 1:** Summary of GLP-1R agonists in addiction models.

Study	GLP-1R Agonist	Substance	Animal Model	Experiment Description	Main Findings
Shirazi et al. (2013) [44]	GLP-1, Ex4	Alcohol	Wistar rats, NMRI mice	Ethanol intake and CPP under intermittent-access; VTA microinjection	Reduced alcohol intake and CPP; VTA GLP-1R activation sufficient
Egecioglu et al. (2013) [45]	Ex4	Alcohol	NMRI mice, Rcc Han Wistar rats	Locomotor assay, microdialysis, PR ethanol self-administration	Blunted ethanol-induced DA release and CPP; reduced intake and PR responding
Vallöf et al. (2019) [46]	Ex4	Alcohol	NMRI mice, Wistar rats	Intra-NTS Ex4; CPP, locomotor and DA response to ethanol	NTS GLP-1R activation reduced alcohol-induced locomotion and CPP; blocked by antagonist
Vallöf et al. (2019) [47]	Ex4	Alcohol	NMRI mice, Wistar rats	NAc/VTA/LDTg site-specific infusions; CPP, intake, gene expression	Region-specific effects: NAc and LDTg suppression; aVTA non-responsive
Colvin et al. (2020) [48]	Ex4	Alcohol	Sprague Dawley rats	Unilateral Ex4 in 8 brain regions; 2-bottle ethanol choice	Reduced alcohol intake in VTA, NAc, LH, DMHipp; no effect in BLA, ArcN, PVN
Aranäs et al. (2023) [49]	Semaglutide	Alcohol	Wistar rats, NMRI mice	Relapse model with alcohol deprivation; DA metabolites, microdialysis	Reduced alcohol and relapse-like drinking; increased DA catabolism
Egecioglu et al. (2013) [45]	Ex4	Nicotine	NMRI mice	Open field, CPP, microdialysis with nicotine	Reduced nicotine-induced locomotion and CPP; blocked DA release in NAc
Tuesta et al. (2017) [50]	Ex4, Sitagliptin	Nicotine	Wild-type and *GLP-1R^−/−^* mice	Circuit mapping of NTS → MHb → IPN; oral self-administration	NTS → MHb → IPN circuit mediates nicotine avoidance; *GLP-1R KO* increases intake
Herman et al. (2023) [51]	Liraglutide	Nicotine	Sprague Dawley rats	IV nicotine SA, extinction, reinstatement, HFD intake	Liraglutide reduced reinstatement and withdrawal-induced hyperphagia
Erreger et al. [52] (2012)	Ex4	Amphetamine	Sprague Dawley rats	Amphetamine-induced locomotion, VTA DA neuron firing	Reduced amphetamine-induced locomotion; decreased VTA DA neuron activity
Graham et al. (2012) [53]	Ex4	Cocaine	C57BL/6J mice	CPP for cocaine with Ex4 pretreatment	Blocked expression of cocaine-induced CPP
Egecioglu et al. (2013) [45]	Ex4	Cocaine, Amphetamine	NMRI mice	Locomotor sensitization, CPP, DA microdialysis	Blunted stimulant-induced DA release and CPP; reduced motivation
Harasta et al. (2015) [54]	Ex4	Cocaine	Conditional *GLP-1R KO* mice	Behavioral sensitization and CPP in *GLP-1R KO* mice	*GLP-1R* in lateral septum required to regulate cocaine-induced behaviors
Reddy et al. (2016) [55]	Ex4	Cocaine	Sprague Dawley rats	DAT function and arachidonic acid signaling in septum	Normalized DAT function and lipid signaling in lateral septum
Schmidt et al. (2016) [56]	Ex4	Cocaine	Sprague Dawley rats	Operant SA, extinction, reinstatement; c-Fos in NAc	Reduced cocaine intake and reinstatement; increased NAc c-Fos
Sirohi et al. (2016) [57]	Liraglutide	Cocaine	Sprague Dawley rats	Cocaine CPP and locomotor testing with liraglutide	Attenuated CPP and locomotor activation by cocaine
Sørensen et al. (2016) [58]	Ex4	Amphetamine	Wild-type and *GLP-1R^−/−^* mice	Amphetamine sensitization and CPP in *GLP-1R^−/−^* vs. *WT* mice	Reduced amphetamine CPP and sensitization in *WT*, not in *GLP-1R^−/−^* mice
Fortin & Roitman (2017) [30]	Ex4	Cocaine	Sprague Dawley rats	Cue-induced phasic DA signaling with voltammetry	Blunted phasic DA response to cocaine cues in NAc
Hernandez et al. (2018) [59]	Ex4	Cocaine	Sprague Dawley rats	Intra-VTA microinjection; cue-induced reinstatement	VTA Ex4 reduced reinstatement; systemic mimicked local effects
Hernandez et al. (2019) [27]	Ex4	Cocaine	Sprague Dawley rats	Electrophysiology in NAc; Ex4 during abstinence	Increased excitability of D1 neurons in NAc; reduced reinstatement
Hernandez et al. (2021) [60]	Ex4	Cocaine	Sprague Dawley rats	Optogenetics/pharmacology in LDTg → VTA GABA projections	LDTg GABA neurons suppressed cocaine seeking via VTA inhibition
Merkel et al. (2025) [61]	Ex4	Cocaine	Sprague Dawley rats	Fiber photometry and RNA-seq in NTS → VTA GABA circuit	NTS → VTA GABA circuit mediates Ex4-induced cocaine suppression
Douton et al. (2021) [62]	Ex4	Heroin	Sprague Dawley rats	Reward devaluation, saccharin-heroin pairing, reinstatement test	Reduced heroin seeking during abstinence; increased OX1R in NAcS
Zhang et al. (2020) [63]	Liraglutide	Oxycodone	Wistar rats	Oxycodone SA under FR and PR, cue reinstatement	Reduced oxycodone intake and reinstatement; decreased CeA activation
Łupina et al. (2020) [64]	Ex4	Morphine	C57BL/6 mice	Naloxone-precipitated withdrawal after morphine, EPM, sucrose preference	Reduced somatic and affective withdrawal; normalized corticosterone
Bornebusch et al. (2019) [65]	Ex4	Morphine	Sprague Dawley rats	CPP, hot plate, tail flick for tolerance; morphine hyperlocomotion	No reduction in morphine CPP or tolerance; limited efficacy observed

Abbreviations: Ex4: exendin 4, CPP: conditioned place preference, DA: dopamine, PR: progressive ratio, VTA: ventral tegmental area, NAc: nucleus accumbens, NTS: nucleus of the solitary tract, LDTg: laterodorsal tegmental nucleus, CeA: central amygdala, EPM: elevated plus maze, SA: self-administration, *KO*: knockout, WT: wild type, FR: fixed ratio, HFD: high-fat diet, OX1R: orexin-1 receptor, ArcN: arcuate nucleus, PVN: paraventricular nucleus, BLA: basolateral amygdala, DMHipp: dorsomedial hippocampus.

**Table 2:** Comparative neurobiological mechanisms of GLP-1 receptor agonists across substance use disorders.

Substance Use Disorder	Key Brain Regions Modulated	Neurotransmitter Systems Affected	Mechanisms of Action	Unique Features
Alcohol Use Disorder (AUD)	VTA, NAc, NTS, LH, DMHipp	Dopamine, GABA	↓ Alcohol-induced DA release in NAc ↑ DA catabolism (MAO-A, COMT) Modulation of reward memory and relapse	Acts on multiple nodes (VTA, NTS, LH) Semaglutide confirmed in NAc by fluorescent labeling
Psychostimulant Use Disorder (Cocaine, Amphetamine)	VTA, NAc, LDTg, LS, MHb-IPN	Dopamine, GABA	↓ Drug-induced DA release ↑ D1-MSN excitability Disruption of cue-induced phasic DA signals	Involves lateral septum and NTS → VTA GABAergic circuits
Opioid Use Disorder (OUD)	NAc shell, CeA, PVN, NTS	Dopamine, Orexin, HPA axis	↓ Cue/drug-induced reinstatement ↓ Fos in CeA↑ OX1R expression ↑ Orexin-1 receptor (OX1R) expression ↓ Withdrawal signs	Modulates affective and somatic withdrawal via HPA normalization

Abbreviations: VTA: ventral tegmental area, NAc: nucleus accumbens, NTS: nucleus tractus solitarius, LH: lateral hypothalamus, DMHipp: dorsomedial hippocampus, LDTg: laterodorsal tegmental nucleus, LS: lateral septum, CeA: central amygdala, PVN: paraventricular nucleus, DA: dopamine, ↓: decreased, ↑: increased, MAO-A: monoamine oxidase A, COMT: catechol-O-methyltransferase, D1-MSN: D1-type medium spiny neuron, OX1R: orexin-1 receptor, HPA: hypothalamic–pituitary–adrenal axis.

**Table 3:** Pharmacokinetic and physicochemical properties of GLP-1 analogues.

GLP-1 Analogue (ATC Code)	Degree of Sequence Homology with Human GLP-1	Half-Life	Molecular Weight	Absorption and Distribution	Metabolism	Excretion	FDA Approval Date	Base
Exenatide (A10BJ01)	53%	2.4 h	4186.6 Da	Tmax 2.5 h Bioavailability 100% Vd 28.3 L	Exenatide is filtered through the glomerulus before being degraded to smaller peptides and amino acids by dipeptidyl peptidase-4, metalloproteases, endopeptidase, amino proteases, and serine proteases	Exenatide is mainly eliminated by glomerular filtration followed by proteolysis before finally being eliminated in the urine. Clearance 9.1 L/h	25 April 2005	Exendin 4
Lixisenatide (A10BJ03)	50%	3 h	4858 Da	Tmax 1–3.5 h Vd 100 L	Catabolized via non-specific proteolytic degradation.	Eliminated via glomerular filtration and proteolytic degradation. Clearance 35 L/h	28 July 2016	Exendin 4
Beinaglutide (A10BJ07)	100%	Not available	3297.6 Da	Not available	Not available		Beinaglutide is under investigation in clinical trial NCT03829891	Human GLP-1
Liraglutide (A10BJ02)	97%	13 h	3751.2 Da	Tmax 11.7 h Bioavailability 55% subcutaneous pathway Vd 13 L	Less sensitive to metabolism than the endogenous GLP-1 and so is more slowly metabolized by dipeptidyl peptidase-4 and neutral endopeptidase to various smaller polypeptides which have not all been structurally determined. A portion of Liraglutide may be completely metabolized to carbon dioxide and water.	6% excreted in urine and 5% excreted in feces. Clearance 1.2 L/h	25 January 2010	Human GLP-1
Dulaglutide	90%	3.75 days (89.9 h) Extended half-life: 5 h	59,669.81 Da	Tmax 24–48 h Bioavailability 65% (subcutaneous injections of single 0.75 mg) and 47% (subcutaneous injections of single 1.5 mg) Vd 3.09 L	Degraded into its component amino acids by general protein catabolism pathways.	Elimination of dulaglutide is expected to occur through degradation to individual amino acids.	Approved in 2014, February 2020 was approved for use in patients with T2DM and multiple cardiovascular risk factors for the prevention of cardiovascular events.	Human GLP-1
Semaglutide (A10BJ06)	94%	168 h	4113.641 Da	Tmax of 56 h Absolute bioavailability is 89% Vd 8 L to 9.4 L. It crosses the placenta in rats.	Semaglutide is cleaved at the peptide backbone, followed by β-oxidation of the fatty acid chain. Chemical structure modifications with sodium N-[8-(2-hydroxybenzoyl) aminocaprylate] or SNAC, an absorption enhancer, render oral semaglutide less susceptible to enzymatic degradation by gastrointestinal DPP-4 enzymes. DPP-4 inactivates semaglutide, truncating the N-terminal segment while NEP hydrolyzes peptide bonds six different metabolites of semaglutide have been identified in human plasma.	This drug is mainly cleared by the kidneys, and is found excreted in both the urine and feces. Clearance: 0.039 L/h. (0.05 L/h in patients with T2DM).	Subcutaneous injection in December 2017. Oral administration in September 2019. In June 2021, it was approved by the FDA for chronic weight management in adults with general obesity or overweight who have at least one weight-related condition. The salt forms of semaglutide (semaglutide sodium and semaglutide acetate) has not been proven to be safe or effective.	Human GLP-1
Albiglutide (A10BJ04)	97%	4–7 days	72,970 Da	Tmax 3 to 5 days post-dosing Vd 11 L	Biotransformation studies have not been performed. Albiglutide is an albumin fusion protein which is catabolized primarily in the vascular endothelium.	Not available Cl 67 mL/h	15 April 2014	Human GLP-1
Taspoglutide	93%	Not available	Not available	Tmax 24 h	Not available	Not available	In September 2010, Roche halted Phase III clinical trials due to incidences of serious hypersensitivity reactions and gastrointestinal side effects.	Not available

**Table 4:** GLP-1 analogues: structural modifications for DPP-4 resistance.

Agent	Structural Modification
Exenatide	Synthetic analogue of exendin-4 (intrinsically DPP-4–resistant); substitution of alanine at position 8 with glycine
Liraglutide	Attachment of a fatty acid chain to Lys^26^ combined with N-terminal modification
Semaglutide	Incorporation of α-aminoisobutyric acid at position 8 and an acylated side chain at Lys^26^, enhancing both DPP-4 resistance and albumin binding, thereby prolonging half-life
Dulaglutide	Fusion to an IgG4 Fc fragment, which provides steric protection against enzymatic degradation
